# Soft Congruence Relations over Rings

**DOI:** 10.1155/2014/541630

**Published:** 2014-04-24

**Authors:** Xiaolong Xin, Wenting Li

**Affiliations:** Department of Mathematics, Northwest University, Xi'an 710127, China

## Abstract

Molodtsov introduced the concept of soft sets, which can be seen as a new mathematical tool for dealing with uncertainty. In this paper, we initiate the study of soft congruence relations by using the soft set theory. The notions of soft quotient rings, generalized soft ideals and generalized soft quotient rings, are introduced, and several related properties are investigated. Also, we obtain a one-to-one correspondence between soft congruence relations and idealistic soft rings and a one-to-one correspondence between soft congruence relations and soft ideals. In particular, the first, second, and third soft isomorphism theorems are established, respectively.

## 1. Introduction

To solve complicated problems in economics, engineering, environmental science, medical science, and social science, methods in classical mathematics are not always successfully used because various uncertainties are typical for these problems. Therefore, there has been a great deal of alternative research and applications in the literature concerning some special tools such as probability theory, fuzzy set theory [1, 2], rough set theory [3, 4], vague set theory [5], and interval mathematics [6]. However, all of these theories have their own difficulties which are pointed out in [7]. In 1999, Molodtsov [7] introduced the concept of soft sets, which can be seen as a new mathematical tool for dealing with uncertainties.

Currently, works on soft set theory are progressing rapidly. Maji et al. [8] discussed the application of soft set theory to a decision making problem. Chen et al. [9] presented a new definition of soft set parametrization reduction and compared this definition to the related concept of attributes reduction in rough set theory. In theoretical aspects, Maji et al. [10] defined and studied several operations on soft sets, and Ali et al. [11] gave some new notions such as restricted intersection, restricted union, restricted difference, and extended intersection of soft sets. Sezgin and Atagün [12] discussed the basic properties of operations on soft sets such as intersection, extended intersection, restricted union, and restricted difference. Aktaş and Çağman [13] compared soft sets to the related concepts of fuzzy sets and rough sets. They also defined the notion of soft groups and derived some related properties. Furthermore, Jun [14] introduced and investigated the notion of soft BCK/BCI-algebras. We also noticed that Feng et al. [15] have already investigated the structure of soft semirings, and Acar et al. [16] have proposed the definition of soft rings and given some properties of soft rings. In [17, 18], Liu et al. have established three isomorphism theorems and fuzzy isomorphism theorems of soft rings. In fact, several researchers have investigated a fuzzy theory in soft structures (see [19]). At the same time, Majumdar and Samanta [20] introduced an idea of soft mapping. Moreover, Ali [21] generalized binary relations and proposed soft binary relations and soft equivalence relations. And Ali et al. [22] introduced algebraic structures of soft sets associated with new operations. In this paper, we extend soft binary relations over sets to soft congruence relations over rings.

The rest of the paper is organized as follows. In Section 2, some basic notions and results about soft sets are given. In Section 3, we introduce soft congruence relations and investigate several related properties. At the same time, a one-to-one correspondence between soft congruence relations and idealistic soft rings over rings is obtained. In Section 4, we obtain that the set of all soft congruence relations associated with some soft operations over a ring can form a complete lattice. And we consider the relations between soft congruence relations and homomorphisms over rings. Also, we establish the first soft isomorphism theorem. In Section 5, we obtain some related results of soft congruence relations of soft rings which is similar to soft congruence relations over rings and set up the second and third soft isomorphism theorems, respectively.

## 2. Preliminaries

In this section, we recall some notions and definitions (see [7, 15, 22, 23]) that will be used in the sequel.

From now on, let *U* be an initial universe and let *E* be a set of parameters. Let *℘*(*U*) denote the power set of *U* and let *A* and *B* be nonempty subsets of *E*.

**Definition 1 (see [7])**

A pair (*F*, *A*) is called a soft set over *U*, where *F* is a mapping given by *F* : *A* → *℘*(*U*).

**Definition 2 (see [7])**

For two soft sets (*F*, *A*) and (*G*, *B*) over a common universe *U*, we say that (*F*, *A*) is a soft subset of (*G*, *B*) if it satisfies

1. *A*⊆*B*;
2. *F*(*α*)⊆*G*(*α*) for all *α* ∈ *A*.

We write $\left(F,A)\tilde{\subset }(G,B\right)$.

In this case, (*G*, *B*) is said to be a soft super set of (*F*, *A*).

**Definition 3 (see [15])**

The AND-operation of two soft sets (*F*, *A*) and (*G*, *B*) over a common universe *U* is the soft set (*H*, *C*) where *C* = *A* × *B* and for all (*α*, *β*) ∈ *C*, *H*(*α*, *β*) = *F*(*α*)∩*G*(*β*). In this case, we write $\left(F,A)\tilde{\wedge }(G,B)=(H,C\right)$.

**Definition 4 (see [15])**

The OR-operation of two soft sets (*F*, *A*) and (*G*, *B*) over a common universe *U* is the soft set (*H*, *C*) where *C* = *A* × *B* and for all (*α*, *β*) ∈ *C*, *H*(*α*, *β*) = *F*(*α*) ∪ *G*(*β*). In this case, we write (*F*, *A*)$\tilde{\vee }$(*G*, *B*) = (*H*, *C*).

**Definition 5 (see [22])**

The extended intersection of two soft sets (*F*, *A*) and (*G*, *B*) over a common universe *U* is the soft set (*H*, *C*) where *C* = *A* ∪ *B* and for all *α* ∈ *C*,

(1) $$\begin{array}{c} H\left(\alpha \right)=\{\begin{array}{cc} F\left(\alpha \right) & \text{if}\;\alpha \in A\setminus B,\\ G\left(\alpha \right) & \text{if}\;\alpha \in B\setminus A,\\ F\left(\alpha \right)\cap G\left(\alpha \right) & \text{if}\;\alpha \in A\cap B. \end{array} \end{array}$$

In this case, we write (*F*, *A*)∩_*ɛ*_(*G*, *B*) = (*H*, *C*).

**Definition 6 (see [22])**

The restricted intersection of two soft sets (*F*, *A*) and (*G*, *B*) over a common universe *U* is the soft set (*H*, *C*) where *C* = *A*∩*B* ≠ *∅* and for all *α* ∈ *C*, *H*(*α*) = *F*(*α*)∩*G*(*α*). In this case, we write (*F*, *A*)∩_*R*_(*G*, *B*) = (*H*, *C*).

If *A*∩*B* = *∅*, then (*F*, *A*)∩_*R*_(*G*, *B*) = *∅*_*∅*_, where *∅*_*∅*_ is the unique soft set over *U* with an empty parameter set.

**Definition 7 (see [22])**

The extended union of two soft sets (*F*, *A*) and (*G*, *B*) over a common universe *U* is the soft set (*H*, *C*) where *C* = *A* ∪ *B* and for all *α* ∈ *C*,

(2) $$\begin{array}{c} H\left(\alpha \right)=\{\begin{array}{cc} F\left(\alpha \right) & \text{if}\;\alpha \in A\setminus B,\\ G\left(\alpha \right) & \text{if}\;\alpha \in B\setminus A,\\ F\left(\alpha \right)\cup G\left(\alpha \right) & \text{if}\;\alpha \in A\cap B. \end{array} \end{array}$$

In this case, we write (*F*, *A*)∪_*ɛ*_(*G*, *B*) = (*H*, *C*).

**Definition 8 (see [22])**

The restricted union of two soft sets (*F*, *A*) and (*G*, *B*) over a common universe *U* is the soft set (*H*, *C*) where *C* = *A*∩*B* ≠ *∅* and for all *α* ∈ *C*, *H*(*α*) = *F*(*α*) ∪ *G*(*α*). In this case, we write (*F*, *A*)∪_*R*_(*G*, *B*) = (*H*, *C*).

If *A*∩*B* = *∅*, then (*F*, *A*)∪_*R*_(*G*, *B*) = *∅*_*∅*_.

In a similar way, we can define the AND-operation, the OR-operation, the extended intersection, the restricted intersection, the extended union, and the restricted union of a family {(*F*_*i*_, *A*_*i*_) | *i* ∈ *I*} of soft sets over *U* as follows in [23].

## 3. Soft Congruence Relations over Rings

In this section, we will introduce the notion of soft congruence relations and investigate several related properties. From now on, 〈*R*, +, ·〉 denotes a ring.

Let (*F*, *A*) be a soft set over *U*. The set Supp⁡(*F*, *A*) = {*α* ∈ *A* | *F*(*α*) ≠ *∅*} is called the support of the soft set (*F*, *A*). A soft set (*F*, *A*) is said to be nonnull if Supp⁡(*F*, *A*)≠*∅* (see [15]).

Let us recall some definitions about soft relations (see [21]) that we will use in the following paragraphs.

**Definition 9**

Let (*ρ*, *A*) be a soft set over *U* × *U*. Then (*ρ*, *A*) is called a soft binary relation over *U*.

**Definition 10**

A soft binary relation (*ρ*, *A*) over *U* is called a soft equivalence relation over *U* if *ρ*(*α*) ≠ *∅* is an equivalence relation on *U* for all *α* ∈ *A*.

**Definition 11**

An equivalence relation *η* over *R* is called a congruence relation on *R* if *aηb* and *cηd* can imply (*a* + *c*)*η*(*b* + *d*) and (*a* · *c*)*η*(*b* · *d*) for all *a*, *b*, *c*, *d* ∈ *R*.

Let us define now a soft congruence relation over *R*.

**Definition 12**

A nonnull soft set (*ρ*, *A*) over *R* × *R* is called a soft congruence relation over *R* if *ρ*(*α*) is a congruence relation on *R* for all ∈Supp⁡(*ρ*, *A*).

If Supp⁡(*ρ*, *A*) = *∅*, then (*ρ*, *A*) is said to be a null soft congruence relation over *R*, denoted by *∅*_*A*_^2^.

Let (*ρ*, *A*) be a soft congruence relation over *R*. If the parameter set *A* is a singleton set, (*ρ*, *A*) is equivalent to a classical congruence relation. If not, (*ρ*, *A*) is a general soft congruence relation. That is, the classical congruence can be considered as a soft congruence relation. Then we can give the following two examples of general soft congruence relations.

**Example 13**

Let 〈*Z*, +, ·〉 be a ring and *A* = *N* ^+^. Let us consider the set-valued function *ρ* : *A* → *℘*(*Z* × *Z*) given by *ρ*(*α*) = {(*x*, *y*) ∈ *Z* × *Z* | *x* ≡ *y*(mod⁡ *α*)} for all *α* ∈ *A*; that is, *ρ*(*α*) is a congruence on module *α*. It is clear that *ρ*(*α*) is a congruence relation on 〈*Z*, +, ·〉. Hence (*ρ*, *A*) is a soft congruence relation over *Z*.

**Example 14**

Let *A* = *R* and let *ρ* : *A* → *℘*(*R* × *R*) be a set-valued function defined by *ρ*(*α*) = {(*x*, *y*) ∈ *R* × *R* | *x* − *y* ∈ 〈*α*〉} for all *α* ∈ *A*, where 〈*α*〉 is the principal ideal generated by *α*. It is easy to verify that *ρ*(*α*) is a congruence relation on *R*. Hence (*ρ*, *A*) is a soft congruence relation over *R*.

**Theorem 15**

Let (*ρ*, *A*) and (*σ*, *B*) be soft congruence relations over *R*. Then

1. $\left(\rho ,A)\tilde{\wedge }(\sigma ,B\right)$ is a soft congruence relation over *R* if it is nonnull;
2. (*ρ*, *A*)∩_*ɛ*_(*σ*, *B*) is a soft congruence relation over *R*;
3. (*ρ*, *A*)∩_*R*_(*σ*, *B*) is a soft congruence relation over *R* if it is nonnull.

**Proof**

(1) By Definition 3, let $\left(\rho ,A)\tilde{\wedge }(\sigma ,B)=(\eta ,C\right)$, where *C* = *A* × *B* and *η*(*α*, *β*) = *ρ*(*α*)∩*σ*(*β*) for all (*α*, *β*) ∈ *C*. Then by the hypothesis, (*η*, *C*) is a nonnull soft set over *R*. If (*α*, *β*)∈Supp⁡(*η*, *C*), then *η*(*α*, *β*) = *ρ*(*α*)∩*σ*(*β*) ≠ *∅*. It follows that the nonempty sets *ρ*(*α*) and *σ*(*β*) are both congruence relations on *R*. Hence *η*(*α*, *β*) is a congruence relation on *R* for all (*α*, *β*) ∈ Supp⁡(*η*, *C*) and so $\left(\rho ,A)\tilde{\wedge }(\sigma ,B)=(\eta ,C\right)$ is a soft congruence relation over *R*.

(2) By Definition 5, let (*ρ*, *A*)∩_*ɛ*_(*σ*, *B*) = (*η*, *C*), where *C* = *A* ∪ *B*, and for all *α* ∈ *C*,

(3) $$\begin{array}{c} \eta \left(\alpha \right)=\{\begin{array}{cc} \rho \left(\alpha \right) & \text{if}\;\alpha \in A\setminus B,\\ \sigma \left(\alpha \right) & \text{if}\;\alpha \in B\setminus A,\\ \rho \left(\alpha \right)\cap \sigma \left(\alpha \right) & \text{if}\;\alpha \in A\cap B. \end{array} \end{array}$$

Let ∈Supp⁡(*η*, *C*). If *α* ∈ *A*∖*B*, then *η*(*α*) = *ρ*(*α*) ≠ *∅* is a congruence relation on *R*; if *α* ∈ *B*∖*A*, then *η*(*α*) = *σ*(*α*) ≠ *∅* is a congruence relation on *R*; and if *α* ∈ *A*∩*B*, *η*(*α*) = *ρ*(*α*)∩*σ*(*α*) ≠ *∅*. Thus *∅* ≠ *ρ*(*α*) and *∅* ≠ *σ*(*α*) are both congruence relations on *R* and so is their intersection. Hence *η*(*α*) is a congruence relation on *R* for all ∈Supp⁡(*η*, *C*). It follows that (*ρ*, *A*)∩_*ɛ*_(*σ*, *B*) = (*η*, *C*) is a soft congruence relation over *R*.

(3) The proof is similar to (1).

**Theorem 16**

Let (*ρ*, *A*) and (*σ*, *B*) be soft congruence relations over *R*. Then

1. $\left(\rho ,A)\tilde{\vee }(\sigma ,B\right)$ is a soft congruence relation over *R* if *ρ*(*α*)⊆*σ*(*β*) or *σ*(*β*)⊆*ρ*(*α*) for all (*α*, *β*) ∈ *A* × *B*;
2. (*ρ*, *A*)∪_*ɛ*_(*σ*, *B*) is a soft congruence relation over *R* if *ρ*(*α*)⊆*σ*(*α*) or *σ*(*α*)⊆*ρ*(*α*) for all *α* ∈ *A* ∪ *B*;
3. (*ρ*, *A*)∪_*R*_(*σ*, *B*) is a soft congruence relation over *R* if *ρ*(*α*)⊆*σ*(*α*) or *σ*(*α*)⊆*ρ*(*α*) for all *α* ∈ *A*∩*B* with *A*∩*B* ≠ *∅*.

**Proof**

(1) By Definition 4, let (*ρ*, *A*)$\tilde{\vee }$(*σ*, *B*) = (*η*, *C*), where *C* = *A* × *B* and *η*(*α*, *β*) = *ρ*(*α*) ∪ *σ*(*β*) for all (*α*, *β*) ∈ *C*. Let (*α*, *β*)∈Supp⁡(*η*, *C*); then *η*(*α*, *β*) = *ρ*(*α*) ∪ *σ*(*β*) ≠ *∅*. Then by the hypothesis, we know that *ρ*(*α*)⊆*σ*(*β*) or *σ*(*β*)⊆*ρ*(*α*) for all (*α*, *β*) ∈ *A* × *B*. It follows that the nonempty sets *ρ*(*α*) and *σ*(*β*) are both congruence relations on *R*. Hence *η*(*α*, *β*) is a congruence relation on *R* for all (*α*, *β*)∈Supp⁡(*η*, *C*) and so (*ρ*, *A*)$\tilde{\vee }$(*σ*, *B*) = (*η*, *C*) is a soft congruence relation over *R*.

(2) By Definition 7, let (*ρ*, *A*)∪_*ɛ*_(*σ*, *B*) = (*η*, *C*), where *C* = *A* ∪ *B*, and for all *α* ∈ *C*,

(4) $$\begin{array}{c} \eta \left(\alpha \right)=\{\begin{array}{cc} \rho \left(\alpha \right) & \text{if}\;\alpha \in A\setminus B,\\ \sigma \left(\alpha \right) & \text{if}\;\alpha \in B\setminus A,\\ \rho \left(\alpha \right)\cup \sigma \left(\alpha \right) & \text{if}\;\alpha \in A\cap B. \end{array} \end{array}$$

Let *α*∈ Supp(*η*, *C*). If *α* ∈ *A*∖*B*, then *η*(*α*) = *ρ*(*α*) ≠ *∅* is a congruence relation on *R*; if *α* ∈ *B*∖*A*, then *η*(*α*) = *σ*(*α*) ≠ *∅* is a congruence relation on *R*; and if *α* ∈ *A*∩*B*, *η*(*α*) = *ρ*(*α*) ∪ *σ*(*α*) ≠ *∅*. Then by the hypothesis, we know that *ρ*(*α*)⊆*σ*(*α*) or *σ*(*α*)⊆*ρ*(*α*) for all *α* ∈ *A* ∪ *B*. Hence *η*(*α*) is a congruence relation on *R* for all *α*∈ Supp(*η*, *C*). It follows that (*ρ*, *A*)∪_*ɛ*_(*σ*, *B*) = (*η*, *C*) is a soft congruence relation over *R*.

(3) The proof is similar to (1).

In a similar way, we can obtain similar properties associated with the AND-operation, the extended intersection, the restricted intersection, the OR-operation, the extended union, and the restricted union of a family {(*ρ*_*i*_, *A*_*i*_) | *i* ∈ *I*} of soft congruence relations over *R*.

Then, we will consider relations between soft congruence relations and idealistic soft rings over rings. In order to do this, we recall the following notions and results.

If there exists an element 0 ∈ *R* such that 0 · *x* = *x* · 0 = 0 and 0 + *x* = *x* + 0 = *x* for all *x* ∈ *R*. Then 0 is called the zero of *R*. From now on, let 0 be the zero of *R*.

**Definition 17 (see [16])**

Let (*F*, *A*) be a nonnull soft set over *R*. Then (*F*, *A*) is called a soft ring over *R*, denoted by $(F,A)\;\tilde{<}\;\;R$, if *F*(*α*) is a subring of *R* for all *α* ∈ *A*.

**Definition 18 (see [16])**

Let (*F*, *A*) be a nonnull soft set over *R*. Then (*F*, *A*) is called an idealistic soft ring over *R* if *F*(*α*) is an ideal of *R* for all ∈Supp⁡(*F*, *A*).

**Lemma 19 (see [24])**

Let *η* be a congruence relation on *R*. Then *I* = 0*η* is an ideal of *R* and (*x*, *y*) ∈ *η*⇔*x* − *y* ∈ *I*. Conversely, let *I* be an ideal of *R* and define (*x*, *y*) ∈ *η*⇔*x* − *y* ∈ *I* in *R*. Then *η* is a congruence relation and 0*η* = *I*.

**Definition 20**

Let (*ρ*, *A*) be a soft congruence relation over *R*. For *x* ∈ *R*, we can consider the set-valued function *xρ* : *A* → *℘*(*R*) given by (*xρ*)(*α*) = *xρ*(*α*) for all *α* ∈ *A*, where *xρ*(*α*) is the congruence class of *x* with respect to *ρ*(*α*). We say that *x*(*ρ*, *A*) is a soft congruence class of *x* with respect to (*ρ*, *A*).

**Definition 21**

Let (*ρ*, *A*) be a soft congruence relation over 〈*R*, +, ·〉 and let *R*/(*ρ*, *A*) = {*xρ*(*α*) | *x* ∈ *R*, *α* ∈ *A*} be an initial universe set. Let us consider the set-valued function ρ* : *A* → *℘*(*R*/(*ρ*, *A*)) given by ρ*(*α*) = {*xρ*(*α*) | *x* ∈ *R*} for all *α* ∈ *A*. We say that (ρ*, *A*) is a soft quotient set of *R*. For all *α* ∈ *A*, 〈ρ*(*α*), +*, ·*〉 is a quotient ring of 〈*R*, +, ·〉 with respect to *ρ*(*α*); we say that (ρ*, *A*) is a soft quotient ring of *R* with respect to the soft congruence relation (*ρ*, *A*). Here, for *x*, *y* ∈ *R*, we have *xρ*(*α*)+*yρ(*α*) = (*x* + *y*)*ρ*(*α*) and (*xρ*(*α*))·*(*yρ*(*α*)) = (*x* · *y*)*ρ*(*α*) for all *α* ∈ *A*.

**Example 22**

Let *R* = {0, *a*, *b*, *c*} be a ring with the operation tables given in (5). For *A* = {0, *a*}, let *ρ* : *A* → *℘*(*R* × *R*) be a set-valued function defined by *ρ*(*α*) = {(*x*, *y*) | *x* + *y* ∈ {0, *α*}} for all *α* ∈ *A*. Then *ρ*(0) = {(0,0), (*a*, *a*), (*b*, *b*), (*c*, *c*)} and *ρ*(*a*) = {(0,0), (*a*, *a*), (*b*, *b*), (*c*, *c*), (0, *a*), (*a*, 0), (*b*, *c*), (*c*, *b*)}, which are both congruence relations on *R*. Hence (*ρ*, *A*) is a soft congruence relation over *R*. And let (ρ*, *A*) be a soft quotient ring of *R* with respect to (*ρ*, *A*).

The operation equations of the ring *R* are as follows:

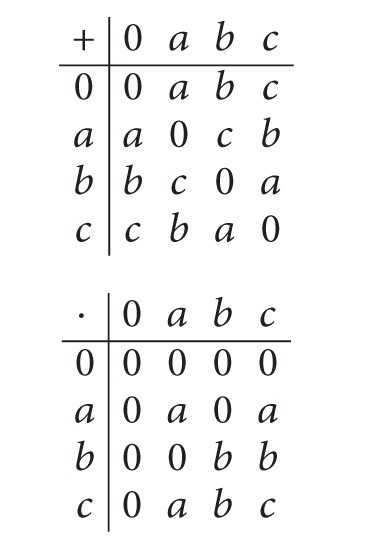
(5)
For *x* ∈ *Z*, we have soft congruence classes:

(6) $$\begin{array}{c} 0\left(\rho ,A\right)=\left\{0\rho \left(0\right),0\rho \left(a\right)\right\}=\left\{\left\{0\right\},\left\{0,a\right\}\right\},\\ a\left(\rho ,A\right)=\left\{a\rho \left(0\right),a\rho \left(a\right)\right\}=\left\{\left\{a\right\},\left\{0,a\right\}\right\}\\ b\left(\rho ,A\right)=\left\{b\rho \left(0\right),b\rho \left(a\right)\right\}=\left\{\left\{b\right\},\left\{b,c\right\}\right\},\\ c\left(\rho ,A\right)=\left\{c\rho \left(0\right),c\rho \left(a\right)\right\}=\left\{\left\{c\right\},\left\{b,c\right\}\right\}. \end{array}$$

Soft quotient ring (ρ*, *A*) is as follows:

(7) $$\begin{array}{c} \rho^{*}\left(0\right)=\left\{0\rho \left(0\right),a\rho \left(0\right),b\rho \left(0\right),c\rho \left(0\right)\right\},\\ \rho^{*}\left(a\right)=\left\{0\rho \left(a\right),a\rho \left(a\right),b\rho \left(a\right),c\rho \left(a\right)\right\}. \end{array}$$

The next two theorems show connections between soft congruence relations and idealistic soft rings over rings.

**Theorem 23**

(1) Let (*ρ*, *A*) be a soft congruence relation over *R*. If (*F*, *A*) = 0(*ρ*, *A*), then (*F*, *A*) is an idealistic soft ring over *R*, and we have *ρ*(*α*) = {(*x*, *y*) ∈ *R* × *R* | *x* − *y* ∈ *F*(*α*)} for all *α* ∈ *A*.

(2) Let (*F*, *A*) be an idealistic soft ring over *R* and consider the set-valued function *ρ* : *A* → *℘*(*R* × *R*) defined by *ρ*(*α*) = {(*x*, *y*) ∈ *R* × *R* | *x* − *y* ∈ *F*(*α*)} for all *α* ∈ *A*. Then (*ρ*, *A*) is a soft congruence relation over *R* and 0(*ρ*, *A*) = (*F*, *A*).

**Proof**

(1) By Definition 12, we know that, for all ∈Supp⁡(*ρ*, *A*), *ρ*(*α*) is a congruence relation on *R*. And according to Lemma 19, we have that *F*(*α*) = 0*ρ*(*α*) is an ideal of *R*, and define (*x*, *y*) ∈ *ρ*(*α*)⇔*x* − *y* ∈ *F*(*α*) in *R*. Since (*F*, *A*) = 0(*ρ*, *A*) is a nonnull soft set, we have that (*F*, *A*) is an idealistic soft ring over *R* by Definition 17 and *ρ*(*α*) = {(*x*, *y*) ∈ *R* × *R* | *x* − *y* ∈ *F*(*α*)} for all *α* ∈ *A*.

(2) By Definition 18, we know that, for all ∈Supp⁡(*F*, *A*), *F*(*α*) is an ideal of *R*. And according to Lemma 19, we have that *ρ*(*α*) is a congruence relation on *R* and 0*ρ*(*α*) = *F*(*α*) for all ∈Supp⁡(*ρ*, *A*). Hence, (*ρ*, *A*) is a soft congruence relation over *R* and 0(*ρ*, *A*) = (*F*, *A*).

Here, we obtain that any soft congruence relation (*ρ*, *A*) over *R* can be represented by the idealistic soft ring generated by (*ρ*, *A*). Also, we observe that any idealistic soft ring (*F*, *A*) over *R* is the soft congruence class of 0 with respect to the soft congruence relation generated by (*F*, *A*).

Summarizing Theorem 23, we have the following facts.

Denote by SC(*R*)^*E*^ the set of all soft congruence relations and by IS(*R*)^*E*^ the set of all idealistic soft rings over *R*. We can establish the following two mappings:

1. *ψ* : SC(*R*)^*E*^ → IS(*R*)^*E*^, *ψ*((*ρ*, *A*)) = (*F*, *A*), where (*F*, *A*) = 0(*ρ*, *A*);
2. *φ* : IS(*R*)^*E*^ → SC(*R*)^*E*^, *φ*((*F*, *A*)) = (*ρ*, *A*), where for all *α* ∈ *A*, we define (*x*, *y*) ∈ *ρ*(*α*)⇔*x* − *y* ∈ *F*(*α*) in *R*.

**Theorem 24**

The above two mappings *ψ* and *φ* are inverse mappings. Then there is a one-to-one correspondence between *SC*(*R*)^*E*^ and *IS*(*R*)^*E*^ over *R*.

## 4. Soft Congruence Relations and Homomorphisms

At the beginning of this section, we study the set of all soft congruence relations associated with some soft operations over a ring which can form a complete lattice.

**Definition 25**

A soft congruence relation (*ρ*, *A*) over *R* is said to be trivial, denoted by *I*_*A*_^2^, if *ρ*(*α*) = {(*x*, *x*) | *x* ∈ *R*} for all *α* ∈ *A*. A soft congruence relation (*ρ*, *A*) over *R* is said to be whole, denoted by *R*_*A*_^2^, if *ρ*(*α*) = {(*x*, *y*) | *x*, *y* ∈ *R*} for all *α* ∈ *A*.

**Example 26**

Let *R* = *Z*/〈4〉 and (*ρ*, *A*_*i*_)_*i*=1,2_ be nonnull soft sets over *R* × *R*. For all *α* ∈ *A*_*i*_, we have *ρ*(*α*) = {(*x*, *y*) ∈ *R* × *R* | *α* · |*x* − *y* | = 0}. For *A*_1_ = {1,3}, it is easy to verify that (*ρ*, *A*_1_) is a soft congruence over *R*, and for all *α* ∈ *A*_1_, *ρ*(*α*) = {(*x*, *x*) | *x* ∈ *R*}. Then (*ρ*, *A*_1_) is the trivial soft congruence over *R*. For *A*_2_ = {0}, it is clear that (*ρ*, *A*_2_) is a soft congruence over *R* and *ρ*(0) = {(*x*, *y*) | *x*, *y* ∈ *R*}. Then (*ρ*, *A*_2_) is the whole soft congruence over *R*.

In a similar way, we can define the whole soft congruence relation with respect to the set of parameters *E* which is called the absolute soft congruence over *R* and simply denoted by *R*_*E*_^2^. And, we will denote by *∅*_*∅*_^2^ the unique soft congruence over *R* with an empty parameter set, which is called the empty soft congruence over *R*.

Now we consider whether the set of all soft congruence relations associated with some soft operations over a ring can form a complete lattice. For the concepts and results of lattices, we can see reference [25].

Let (*ρ*, *A*) be a nonnull soft set over *R* × *R*. We call the smallest soft congruence relation which is the soft super set of (*ρ*, *A*) to be a soft congruence relation generated by (*ρ*, *A*), denoted by [(*ρ*, *A*)], namely, the restricted intersection of the family of all soft congruence relations over *R* which are soft super sets of (*ρ*, *A*). In this case, we write $\left[\left(\rho ,A\right)\right]=\cap_{R}\left\{\left(\sigma ,B\right)|\left(\rho ,A\right)\tilde{\subset }\left(\sigma ,B\right),\left(\sigma ,B\right)\in \text{SC}{\left(R\right)}^{E}\right\}$.

Let (*ρ*, *A*) and (*σ*, *B*) ∈ SC(*R*)^*E*^. We denote (*ρ*, *A*)∨_*ɛ*_(*σ*, *B*) = [(*ρ*, *A*)∪_*ɛ*_(*σ*, *B*)]. Then $\left(\text{SC}{\left(R\right)}^{E},\tilde{\subset },\vee_{ɛ},\cap_{R}\right)$ is a complete lattice, where *R*_*E*_^2^ and *∅*_*∅*_^2^ are the greatest element and the least element of SC(*R*)^*E*^, respectively.

In a similar way, denote by SC(*R*)_*A*_ the set of all those soft congruence relations defined over *R* with a fixed parameter set *A*. Then $\left(\text{SC}{\left(R\right)}_{A},\tilde{\subset },\vee_{ɛ},\cap_{R}\right)$ is a complete lattice, where *R*_*A*_^2^ and *∅*_*A*_^2^ are the greatest element and the least element of SC(*R*)_*A*_, respectively.

Next, we will consider the relations between soft congruence relations and homomorphisms over rings.

**Lemma 27**

Let *f* : *R* → *R*′ be a ring epimorphism and let *η* be a congruence relation on *R*. Define *f*(*η*) = {(*f*(*x*), *f*(*y*)) ∈ *R*′ × *R*′ | *xηy*}; then *f*(*η*) is a congruence relation on *R*′.

**Proof**

For all *x*′, *y*′ ∈ *R*, and (*x*′, *y*′) ∈ *f*(*η*), since *f* is a ring epimorphism, there exists *x*, *y* ∈ *R*, such that *x*′ = *f*(*x*), *y*′ = *f*(*y*), and *xηy*. It is easy to verify that *f*(*η*) is a congruence relation on *R*′.

Let *f* : *R* → *R*′ be a mapping of rings and let (*F*, *A*) be a soft set over *R*. Then we can define a soft set (*f*(*F*), *A*) over *R*′ where *f*(*F*) : *A* → *℘*(*R*′) is defined as *f*(*F*)(*α*) = *f*(*F*(*α*)) for all *α* ∈ *A*. Here, by definition, we see that Supp⁡(*f*(*F*), *A*) = Supp⁡(*F*, *A*).

**Lemma 28**

Let *f* : *R* → *R*′ be a ring epimorphism. If (*ρ*, *A*) is a soft congruence relation over *R*, then (*f*(*ρ*), *A*) is a soft congruence relation over *R*′, where *f*(*ρ*)(*α*) = {(*f*(*x*), *f*(*y*)) ∈ *R*′ × *R*′ | (*x*, *y*) ∈ *ρ*(*α*)} for all *α* ∈ *A*.

**Proof**

Note first that (*f*(*ρ*), *A*) is a nonnull soft set since (*ρ*, *A*) is a soft congruence relation over *R*, which is a nonnull soft set by Definition 11. For all *α* ∈ Supp⁡(*f*(*ρ*), *A*), we have *f*(*ρ*)(*α*) = *f*(*ρ*(*α*)) ≠ *∅*. Then the nonempty set *ρ*(*α*) is a congruence relation on *R*, and so we deduce that its onto homomorphic image *f*(*ρ*(*α*)) is a congruence on *R*′. Hence *f*(*ρ*)(*α*) is a congruence on *R*′ for all ∈Supp⁡(*f*(*ρ*), *A*). That is, (*f*(*ρ*), *A*) is a soft congruence relation over *R*′.

**Lemma 29**

Let *f* : *R* → *R*′ be a ring epimorphism and let (*ρ*, *A*) be a soft congruence relation over *R*, where *f*(*ρ*)(*α*) = {(*f*(*x*), *f*(*y*)) ∈ *R*′ × *R*′ | (*x*, *y*) ∈ *ρ*(*α*)} for all *α* ∈ *A*.

1. If (*ρ*, *A*) is trivial, then (*f*(*ρ*), *A*) is the trivial soft congruence relation over *R*′.
2. If (*ρ*, *A*) is whole, then (*f*(*ρ*), *A*) is the whole soft congruence relation over *R*′.

**Proof**

(1) By Definition 25, we know that, for all *α* ∈ *A*, *ρ*(*α*) = {(*x*, *x*) | *x* ∈ *R*}. Since *f* is a ring epimorphism, we have that *f*(*ρ*)(*α*) = *f*(*ρ*(*α*)) = {(*x*′, *x*′) | *x*′ = *f*(*x*), *x* ∈ *R*} for all *α* ∈ *A*. So, (*f*(*ρ*), *A*) is the trivial soft congruence relation over *R*′ by Lemma 28.

(2) By Definition 25, we know that, for all *α* ∈ *A*, *ρ*(*α*) = {(*x*, *y*) | *x*, *y* ∈ *R*}. Since *f* is a ring epimorphism, we have that *f*(*ρ*)(*α*) = {(*x*′, *y*′) | *x*′ = *f*(*x*), *y*′ = *f*(*y*), (*x*, *y*) ∈ *ρ*(*α*), *x*, *y* ∈ *R*} for all *α* ∈ *A*. It follows from Lemma 28 that (*f*(*ρ*), *A*) is the whole soft congruence relation over *R*′.

This completes the proof.

Let us define now some definitions about soft quotient rings that we will use in the following paragraphs.

**Definition 30**

Let (*ρ*, *A*) be a soft congruence relation over *R*; we have that (*F*, *A*) = 0(*ρ*, *A*) is an idealistic soft ring over *R* by Theorem 23, and for all *α* ∈ *A*, *xρ*(*α*) = *x* + *F*(*α*). Let *R*/(*F*, *A*) = {*x* + *F*(*α*) | *x* ∈ *R*, *α* ∈ *A*} be an initial universe set and consider the set-valued function F* : *A* → *℘*(*R*/(*F*, *A*)) given by F*(*α*) = {*x* + *F*(*α*) | *x* ∈ *R*} for all *α* ∈ *A*. We say that (F*, *A*) is a soft quotient ring of *R* with respect to the idealistic soft ring (*F*, *A*), where 〈F*(*α*), +*, ·*〉 is a quotient ring of 〈*R*, +, ·〉 with respect to an ideal *F*(*α*).

**Example 31**

Let (*F*, *A*) be an idealistic soft ring over 〈*Z*, +, ·〉 and *A* = *N* ^+^. For all *α* ∈ *A*, we have *F*(*α*) = {*αx* | *x* ∈ *Z*}⊲*Z*. Let *Z*/(*F*, *A*) = {*x* + *F*(*α*) | *x* ∈ *Z*, *α* ∈ *A*} be an initial universe set and consider the set-valued function F* : *A* → *℘*(*Z*/(*F*, *A*)) given by F*(*α*) = *Z*/*F*(*α*) = {0 + *F*(*α*), 1 + *F*(*α*),…, (*α* − 1) + *F*(*α*)} = *Z*/〈*α*〉 for all *α* ∈ *A*; it is clear that F*(*α*) is a quotient ring of *Z* with respect to *F*(*α*). Then (F*, *A*) is a soft quotient ring of *Z* with respect to (*F*, *A*).

**Definition 32**

Let (F*, *A*) be a soft quotient ring of *R* with respect to an idealistic soft ring (*F*, *A*) and let (G*, *B*) be a soft quotient ring of *R*′ with respect to an idealistic soft ring (*G*, *B*), respectively. We say that (F*, *A*) is soft homomorphic to (G*, *B*), denoted by (F*, *A*)~_*Q*_(G*, *B*), if, for all *α* ∈ *A*, there exists *β* ∈ *B* such that a mapping *f* : F*(*α*) → G*(*β*) is a homomorphism; that is, F*(*α*) ~ G*(*β*).

If *f* is an isomorphism, then we say that (F*, *A*) is soft isomorphic to (G*, *B*), which is denoted by (F*, *A*)≃_*Q*_(G*, *B*).

Finally, we establish the first soft isomorphism theorem. In order to do this, we need to introduce some relative notions and results. Note that, if *S* is a subring of *R*, we write *S* ≤ *R*; if *H* is an ideal of *R*, we write *H*⊲*R*.

**Definition 33**

Let (*F*, *A*) and (*G*, *B*) be soft sets over a common universe *U*. (*F*, *A*) is said to be contained by (*G*, *B*), denoted by $\left(F,A){\tilde{\subset }}_{C}(G,B\right)$ if, for all *α* ∈ *A*, there exists *β* ∈ *B* such that *F*(*α*)⊆*G*(*β*).

**Definition 34**

Two soft sets (*F*, *A*) and (*G*, *B*) over a common universe *U* are said to be soft set equal, denoted by (*F*, *A*) = (*G*, *B*), if it satisfies the following:

1. for all *α* ∈ *A*, ∃*β* ∈ *B* such that *F*(*α*) = *G*(*β*);
2. for all *b* ∈ *B*, ∃*a* ∈ *A* such that *G*(*b*) = *F*(*a*).

**Example 35**

Let *U* = {*x*, *y*, *z*, *u*, *v*}, *A* = {*α*, *α*′}, and *B* = {*β*, *β*′}. Let *F* : *A* → *℘*(*R*) and *G* : *B* → *℘*(*R*) be set-valued functions defined as follows: *F*(*α*) = {*x*, *z*}, *F*(*α*′) = {*u*, *v*}, *G*(*β*) = {*u*, *v*}, and *G*(*β*′) = {*x*, *z*}. Therefore, (*F*, *A*) and (*G*, *B*) are soft set equal.

**Lemma 36**

Let *f* : *R* → *R*′ be a ring epimorphism.

1. Let *ρ* : *A*→*℘*(*R* × *R*) be a set-valued function given by *ρ*(*α*) = {(*x*, *y*) | *x*, *y* ∈ *R*, *f*(*x*) = *f*(*y*)} for all *α* ∈ *A*. Then (*ρ*, *A*) is a soft congruence relation over *R*.
2. Let *F* : *A* → *℘*(*R*) be a set-valued function given by *F*(*α*) = Ker*f* = {*x* ∈ *R* | *f*(*x*) = 0} for all *α* ∈ *A*. Then (*F*, *A*) is an idealistic soft ring over *R*.

**Lemma 37 (see [24] first isomorphism theorem)**

Let *f* : *R* → *R*′ be a ring epimorphism. Let *S* = {*H* | *H* ≤ *R*, *H*⊇*K* = Ker*f*} and *T* = {*H*′ | *H*′ ≤ *R*′}. Then, for *H* ∈ *S*, we make *H* correspond to *Hf* = {*f*(*x*) | *x* ∈ *H*}, which is a bijective mapping between *S* and *T*. And *H*⊲*R*⇔*Hf*⊲*R*′; here, *R*/*H*≅*R*′/*Hf*.

**Theorem 38 (first soft isomorphism theorem)**

Let *f* : *R* → *R*′ be a ring epimorphism and consider an idealistic soft ring (*F*, *A*) over *R* defined as Lemma 36(2). Let $SR(R)=\{\left(G,B\right)|(G,B)\tilde{<}R$, $\left(\text{F},A){\tilde{\subset }}_{C}(G,B)\right\}$, and $SR(R^{\prime })=\{(H,C)|\left(H,C\right)\tilde{<}R^{\prime }\}$.

1. For (*G*, *B*) ∈ *SR*(*R*), let *f*(*G*) : *B* → *℘*(*R*′) be a set-valued function given by *f*(*G*)(*α*) = *f*(*G*(*α*)) = {*f*(*x*) | *x* ∈ *G*(*α*)} for all *α* ∈ *B*. We make (*G*, *B*) correspond to (*f*(*G*), *B*), which is a bijective mapping between *SR*(*R*) and *SR*(*R*′).
2. (*G*, *B*) is an idealistic soft ring over *R* if and only if (*f*(*G*), *B*) is an idealistic soft ring over *R*′. And (G*, *B*)≃_*Q*_(*f*(*G*)*, *B*), where (G*, *B*) is a soft quotient ring of *R* with respect to (*G*, *B*), and (*f*(*G*)*, *B*) is a soft quotient ring of *R*′ with respect to (*f*(*G*), *B*).

**Proof**

(1) Let (*G*_1_, *B*_1_), (*G*_2_, *B*_2_) ∈ SR(*R*) if (*f*(*G*_1_), *B*_1_) = (*f*(*G*_2_), *B*_2_); that is, for all *α* ∈ *B*_1_, ∃*β* ∈ *B*_2_ such that *f*(*G*_1_)(*α*) = *f*(*G*_2_)(*β*) and for all *b* ∈ *B*_2_, ∃*a* ∈ *B*_1_ such that *f*(*G*_2_)(*b*) = *f*(*G*_1_)(*a*), but by Lemma 37, we have *G*_1_(*α*) = *G*_2_(*β*) and *G*_2_(*b*) = *G*_1_(*a*), which means that (*G*_1_, *B*_1_) = (*G*_2_, *B*_2_). Thus it is injection. Let (*G*, *B*) ∈ SR(*R*); then we have, for all *α* ∈ *B*, *G*(*α*) ≤ *R* and its homomorphism image *f*(*G*)(*α*) = *f*(*G*(*α*)) ≤ *R*′, so $\left(f\left(G\right),B\right)\tilde{<}R^{\prime }$; that is, (*f*(*G*), *B*) ∈ SR(*R*′). Let (*f*(*G*), *B*) ∈ SR(*R*′); then we have for all *α* ∈ *B*, *f*(*G*)(*α*) ≤ *R*′. Define *G*(*α*) = {*x* ∈ *R* | *f*(*x*) ∈ *f*(*G*)(*α*)} for all *α* ∈ *B*; then *G*(*α*) ≤ *R*. And for all *a* ∈ *A*, ∃*α* ∈ *B*, Ker*f* = *F*(*a*)⊆*G*(*α*). So $(G,B)\tilde{<}R$ and $\left(F,A){\tilde{\subset }}_{C}(G,B\right)$; that is, (*G*, *B*) ∈ SR(*R*). Thus it is surjection. Therefore, we make (*G*, *B*) correspond to (*f*(*G*), *B*), which is a bijective mapping between SR(*R*) and SR(*R*′).

(2) (*G*, *B*) is an idealistic soft ring over *R*⇔G(*α*)⊲*R* (for all *α* ∈ *B*)⇔ (by Lemma 37) *f*(*G*(*α*))⊲*R*′  (for all *α* ∈ *B*)⇔(*f*(*G*), *B*) is an idealistic soft ring over *R*′. From Lemma 37, G*(*α*)≅*f*(*G*)*(*α*) for all *α* ∈ *B*, which means that (G*, *B*)≃_*Q*_(*f*(*G*)*, *B*) by Definition 32.

**Example 39**

Let *f* : *Z* → *Z*/〈6〉 be a ring epimorphism, *A* = *Z* and *B* = *C* = {*α* | *α*  is  divisor  of  6}. Consider the set-valued function *F* : *A* → *℘*(*Z*) given by *F*(*α*) = 6*Z*⊲*Z*. Then (*F*, *A*) is an idealistic soft ring over *Z*. Let $\text{SR}(Z)=\{\left(G,B\right)|(G,B)\tilde{<}Z$, $\left(F,A){\tilde{\subset }}_{C}(G,B)\right\}$. Then for (*G*, *B*) ∈ SR(*Z*), we have *G*(*β*) = *βZ*. And let $\text{SR}(Z/\langle 6\rangle )=\{\left(H,C\right)|(H,C)\tilde{<}Z/\langle 6\rangle \}$. Then for (*H*, *C*) ∈ SR(*Z*/〈6〉), we have *H*(*c*) = *cZ*/〈6〉.

1. Let *B* = *C* = {1,2, 3,6} and let *f*(*G*) : *B* → *℘*(*R*′) be a set-valued function given by *f*(*G*)(*β*) = *βZ*/〈6〉 for all *β* ∈ *B*. Then, (*f*(*G*), *B*) ∈ SR(*Z*/〈6〉). It is clear that we make (*G*, *B*) correspond to (*f*(*G*), *B*), which is a bijective mapping between SR(*Z*) and SR(*Z*/〈6〉).
2. Let *B* = {2,3} and G*(*β*) = {*x* + *G*(*β*) | *x* ∈ *Z*} = *Z*/〈*β*〉. Then (*G*, *B*) is an idealistic soft ring over *R* iff *G*(*β*) = *βZ*⊲*Z* (for all *β* ∈ *B*), *f*(*G*(*β*)) = *βZ*/〈6〉⊲*Z*/〈6〉 (for all *β* ∈ *B*), and (*f*(*G*), *B*) is an idealistic soft ring over *Z*/〈6〉. It is clear that (G*, *B*) is a soft quotient ring of *Z* with respect to (*G*, *B*), and (*f*(*G*)*, *B*) is a soft quotient ring of *Z*/〈6〉 with respect to (*f*(*G*), *B*). Let *β* ∈ *B*; we have G*(*β*) = {*x* + *G*(*β*) | *x* ∈ *Z*} = *Z*/〈*β*〉 and *f*(*G*)*(*β*) = {*x* + *f*(*G*)(*β*) | *x* ∈ *Z*/〈6〉} = (*Z*/〈6〉)/(*βZ*/〈6〉). Note that *Z*/〈2〉≅(*Z*/〈6〉)/(2*Z*/〈6〉) and *Z*/〈3〉≅(*Z*/〈6〉)/(3*Z*/〈6〉); we have (G*, *B*)≃_*Q*_(*f*(*G*)*, *B*) by Definition 32.

## 5. Soft Congruence Relation of Soft Rings

In this section, we study internal connections between soft congruence relations and soft ideals of soft rings and obtain the second and third soft isomorphism theorems. In order to do this, we recall the following notions.

**Definition 40 (see [16])**

Let (*F*, *A*) and (*G*, *B*) be soft rings over *R*. Then (*G*, *B*) is called a soft subring of (*F*, *A*), denoted by $\left(G,B)\tilde{\leq }(F,A\right)$, if it satisfies the following:

1. *B*⊆*A*;
2. *G*(*α*) is a subring of *F*(*α*) for all *α*∈Supp⁡(*G*, *B*).

**Definition 41 (see [16])**

Let (*F*, *A*) be a soft ring over *R*. A nonnull soft set (*G*, *B*) over *R* is called a soft ideal of (*F*, *A*), denoted by $\left(G,B)\tilde{⊲}(F,A\right)$, if it satisfies the following:

1. *B*⊆*A*;
2. *G*(*α*) is an ideal of *F*(*α*) for all *α*∈Supp⁡(*G*, *B*).

**Definition 42**

Let (*F*, *A*) be a soft ring over *R*. A nonnull soft set (*ρ*, *B*) over *R* × *R* is called a soft binary relation of (*F*, *A*) if it satisfies the following:

1. *B*⊆*A*;
2. *ρ*(*α*) is a binary relation of *F*(*α*) for all *α* ∈ *B*.

**Definition 43**

Let (*F*, *A*) be a soft ring over *R*. A soft binary relation (*ρ*, *B*) of (*F*, *A*) is called a soft congruence relation of (*F*, *A*) if *ρ*(*α*) is a congruence relation on *F*(*α*) for all ∈Supp⁡(*ρ*, *B*).

The next theorem shows connections between soft congruence relations and soft ideals of soft rings.

**Theorem 44**

Let (*F*, *A*) be a soft ring over *R*. Then we have the following.

1. Let (*ρ*, *B*) be a soft congruence relation of (*F*, *A*). If (*G*, *B*) = 0(*ρ*, *B*), then (*G*, *B*) is a soft ideal of (*F*, *A*), and we have *ρ*(*α*) = {(*x*, *y*) | *x*, *y* ∈ *F*(*α*), *x* − *y* ∈ *G*(*α*)} for all *α* ∈ *B*.
2. Let (*G*, *B*) be a soft ideal of (*F*, *A*) and consider a soft binary relation (*ρ*, *B*) of (*F*, *A*) defined by *ρ*(*α*) = {(*x*, *y*) | *x*, *y* ∈ *F*(*α*), *x* − *y* ∈ *G*(*α*)} for all *α* ∈ *B*. Then (*ρ*, *B*) is a soft congruence relation of (*F*, *A*), and 0(*ρ*, *B*) = (*G*, *B*).

**Proof**

(1) By Definition 43, we know that, for all ∈Supp⁡(*ρ*, *B*), *ρ*(*α*) is a congruence relation on a ring *F*(*α*). Since (*G*, *B*) = 0(*ρ*, *B*), we have *G*(*α*) = 0*ρ*(*α*)⊲*F*(*α*), and *ρ*(*α*) = {(*x*, *y*) | *x*, *y* ∈ *F*(*α*), *x* − *y* ∈ *G*(*α*)} for all *α* ∈ *B* by Lemma 19. Since (*G*, *B*) is a non-null soft set, we have $\left(G,B)\tilde{⊲}(F,A\right)$.

(2) By Definition 41, we know for all ∈Supp⁡(*G*, *B*), *G*(*α*)⊲*F*(*α*). By the hypothesis, we know that *ρ*(*α*) is a congruence relation on *F*(*α*) and 0*ρ*(*α*) = *G*(*α*) by Lemma 19. Then (*ρ*, *B*) is a soft congruence relation of (*F*, *A*) and 0(*ρ*, *B*) = (*G*, *B*).

Here, we obtain that any soft congruence relation (*ρ*, *B*) of a soft ring (*F*, *A*) can be represented by the soft ideal generated by (*ρ*, *B*). Also, we observe that any soft ideal (*G*, *B*) of (*F*, *A*) is the soft congruence class of 0 with respect to the soft congruence relation (*ρ*, *B*) generated by (*G*, *B*).

Summarizing Theorem 44, we have the following facts.

Denote by SC((*F*, *A*))^*E*^ the set of all soft congruence relations and by SI((*F*, *A*))^*E*^ the set of all soft ideals of a soft ring (*F*, *A*). We can establish the following two mappings:

1. *ψ* : SC((*F*, *A*))^*E*^ → SI((*F*, *A*))^*E*^, *ψ*((*ρ*, *B*)) = (*G*, *B*), where (*G*, *B*) = 0(*ρ*, *B*);
2. *φ* : SI((*F*, *A*))^*E*^ → SC((*F*, *A*))^*E*^, *φ*((*G*, *B*)) = (*ρ*, *B*), where, for all *α* ∈ *B*, we define (*x*, *y*) ∈ *ρ*(*α*) in *F*(*α*), which is equivalent to *x* − *y* ∈ *G*(*α*).

**Theorem 45**

The above two mappings *ψ* and *φ* are inverse mappings. Then there is a one-to-one correspondence between *SC*((*F*, *A*))^*E*^ and *SI*((*F*, *A*))^*E*^ of (*F*, *A*).

**Definition 46**

Let (*F*, *A*) and (*G*, *B*) be soft rings over *R*. We say that (*G*, *B*) is a generalized soft subring of (*F*, *A*), denoted by $\left(G,B){\tilde{\leq }}_{G}(F,A\right)$, if, for all *β* ∈ *B*, there exists *α* ∈ *A* such that *G*(*β*) is a subring of *F*(*α*).

**Definition 47**

Let (*F*, *A*) and (*G*, *B*) be soft rings over *R*. We say that (*G*, *B*) is a generalized soft ideal of (*F*, *A*), denoted by $\left(G,B){\tilde{⊲}}_{G}(F,A\right)$, if, for all *β* ∈ *B*, there exists *α* ∈ *A* such that *G*(*β*) is an ideal of *F*(*α*).

Here, we obtain that a soft ideal of soft ring must be a generalized soft ideal of soft ring.

**Definition 48**

Let (*F*, *A*) be a generalized soft ideal of a soft ring (*G*, *B*) which is over 〈*R*, +, ·〉 and let (*G*, *B*)/(*F*, *A*) = {*x* + *F*(*α*) | (*α*, *β*) ∈ *A*×_∗_ *B*, *x* ∈ *G*(*β*)} be an initial universe set, where *A*×_∗_ *B* = {(*α*, *β*) ∈ *A* × *B* | *F*(*α*)⊲*G*(*β*)}. Let us consider the set-valued function *F*_∗_ : *A*×_∗_ *B* → *℘*((*G*, *B*)/(*F*, *A*)) given by *F*_∗_(*α*, *β*) = {*x* + *F*(*α*) | *x* ∈ *G*(*β*)} for all (*α*, *β*) ∈ *A*×_∗_ *B*. We say that (*F*_∗_, *A*×_∗_ *B*) is a generalized soft quotient ring of soft ring (*G*, *B*) with respect to the generalized soft ideal (*F*, *A*), where 〈*F*_∗_(*α*, *β*), +_∗_, ·_∗_〉 is a quotient ring of ring 〈*G*(*β*), +, ·〉 with respect to *F*(*α*).

**Example 49**

Let *R* = *B* = *Z*/〈4〉 = {0,1, 2,3} and *A* = {0,1, 2}. Let us consider the set-valued function *G* : *B* → *℘*(*R*) given by *G*(*β*) = {*x* ∈ *R* | *x* · *β* = {0,2}}. Then *G*(0) = *R*, *G*(1) = {0,2}, *G*(2) = *Z*/〈4〉, and *G*(3) = {0,2}. As we see, all these sets are subrings of *R*. Hence, (*G*, *B*) is a soft ring over *R*. On the other hand, consider the function *F* : *A* → *℘*(*R*) given by *F*(*α*) = {*x* ∈ *R* | *x* · *α* = 0}. As we see,

(8) F(0)=R, then  F(0)⊲G(0), F(0)⊲G(2);F(1)={0}, then  F(1)⊲G(0), F(1)⊲G(1),F(1)⊲G(2), F(1)⊲G(3);F(2)={0,2}, then  F(2)⊲G(0), F(2)⊲G(1),F(2)⊲G(2), F(2)⊲G(3).

Hence, (*F*, *A*) is a generalized soft ideal of (*G*, *B*). For all (*α*, *β*) ∈ *A* × *B*, where *F*(*α*)⊲*G*(*β*), *F*_∗_(*α*, *β*) = {*x* + *F*(*α*) | *x* ∈ *G*(*β*)}; we have

(9) F∗(0,0)=F∗(0,2)={0+F(0),1+F(0),2+F(0),3+F(0)};F∗(1,0)=F∗(1,2)={0+F(1),1+F(1),2+F(1),3+F(1)};F∗(1,1)=F∗(1,3)={0+F(1),2+F(1)};F∗(2,0)=F∗(2,2)={0+F(2),1+F(2),2+F(2),3+F(2)};F∗(2,1)=F∗(2,3)={0+F(2),2+F(2)}.

As we see, *F*_∗_(*α*, *β*) are quotient rings of ring 〈*G*(*β*), +, ·〉 with respect to *F*(*α*). Thus (*F*_∗_, *A*×_∗_ *B*) is a generalized soft quotient ring of soft ring (*G*, *B*) with respect to the generalized soft ideal (*F*, *A*).

**Definition 50**

Let (*F*, *A*) and (*G*, *B*) be generalized soft ideals of soft rings (*F*′, *A*′) and (*G*′, *B*′), respectively. Suppose (*F*_∗_, *A*×_∗_ *A*′) is a generalized soft quotient ring of (*F*′, *A*′) with respect to (*F*, *A*), and (*G*_∗_, *B*×_∗_ *B*′) is a generalized soft quotient ring of (*G*′, *B*′) with respect to (*G*, *B*). We say that (*F*_∗_, *A*×_∗_ *A*′) is soft homomorphic to (*G*_∗_, *B*×_∗_ *B*′), denoted by (*F*_∗_, *A*×_∗_ *A*′)∼_*G*_(*G*_∗_, *B*×_∗_ *B*′), if, for all (*α*, *α*′) ∈ *A*×_∗_ *A*′, there exists (*β*, *β*′) ∈ *B*×_∗_ *B*′ such that a mapping *f* : *F*_∗_(*α*, *α*′) → *G*_∗_(*β*, *β*′) is a homomorphism; that is, *F*_∗_(*α*, *α*′) ~ *G*_∗_(*β*, *β*′).

If *f* is an isomorphism, then we say that (*F*_∗_, *A*×_∗_ *A*′) is soft isomorphic to (*G*_∗_, *B*×_∗_ *B*′), which is denoted by (*F*_∗_, *A*×_∗_ *A*′)≃_*G*_(*G*_∗_, *B*×_∗_ *B*′).

**Definition 51 (see[16])**

Let (*F*, *A*) and (*G*, *B*) be soft rings over the rings *R* and *R*′, respectively. Let *f* and *g* be two mappings. The pair (*f*, *g*) is called a soft ring homomorphism if the following conditions are satisfied:

1. *f* is a ring epimorphism;
2. *g* is surjective;
3. *f*(*F*(*α*)) = *G*(*g*(*α*)) for all *α* ∈ *A*.

If we have a soft ring homomorphism between (*F*, *A*) and (*G*, *B*), (*F*, *A*) is said to be soft homomorphic to (*G*, *B*), denoted by (*F*, *A*)~(*G*, *B*). In addition, if *f* is a ring isomorphism and *g* is a bijective mapping, then (*f*, *g*) is called a soft ring isomorphism. In this case, we say that (*F*, *A*) is soft isomorphic to (*G*, *B*), denoted by (*F*, *A*)≃(*G*, *B*).

**Definition 52**

The basic sum of two soft rings (*F*, *A*) and (*G*, *B*) over *R* is the soft set (*H*, *C*) where *C* = *A* × *B* and for all (*α*, *β*) ∈ *C*, *H*(*α*, *β*) = *F*(*α*) + *G*(*β*). In this case, we write (*F*, *A*)+(*G*, *B*) = (*H*, *C*).

**Lemma 53**

Let (*F*, *A*) and (*G*, *B*) be soft rings over rings *R* and *R*′, respectively. Let (*f*, *g*) be a soft ring homomorphism between (*F*, *A*) and (*G*, *B*), and *A*′⊆*A*.

1. Let us consider a soft binary relation (*ρ*, *A*′) of (*F*, *A*) defined by *ρ*(*α*) = {(*x*, *y*) | *x*, *y* ∈ *F*(*α*), *f*(*x*) = *f*(*y*)} for all *α* ∈ *A*′. Then (*ρ*, *A*′) is a soft congruence relation of (*F*, *A*).
2. Let *F*′ : *A*′ → *℘*(*R*) be a set-valued function given by *F*′(*α*) = Ker*f*(*F*(*α*)) = {*x* ∈ *F*(*α*) | *f*(*x*) = 0} for all *α* ∈ *A*′. Then (*F*′, *A*′) is a soft ideal of (*F*, *A*).

**Theorem 54 (second soft isomorphism theorem)**

Let (*F*, *A*) and (*G*, *B*) be soft rings over rings *R* and *R*′, respectively. Let (*f*, *g*) be a soft ring epimorphism between (*F*, *A*) and (*G*, *B*), and consider a soft ideal (*F*′, *A*′) of (*F*, *A*) defined as Lemma 53(2). Denote $SS((F,A))=\{\left(F_{1},A_{1}\right)|(F_{1},A_{1})\tilde{\leq }(F,\text{A}),(F^{\prime },A^{\prime }){\tilde{\subset }}_{C}(F_{1},A_{1})\}$ and $SS((G,B))=\{(G_{1},B_{1})|(G_{1},B_{1}){\tilde{\leq }}_{G}(G,B)\}$.

1. Let (*F*_1_, *A*_1_) ∈ *SS*((*F*, *A*)) and consider the set-valued function *f*(*F*_1_) : *A*_1_ → *℘*(*R*′) given by *f*(*F*_1_)(*α*) = *f*(*F*_1_(*α*)) = {*f*(*x*) | *x* ∈ *F*_1_(*α*)} for all *α* ∈ *A*_1_. Then we make (*F*_1_, *A*_1_) correspond to (*f*(*F*_1_), *A*_1_), which is a bijective mapping between *SS*((*F*, *A*)) and *SS*((*G*, *B*)).
2. (*F*_1_, *A*_1_) is a soft ideal of (*F*, *A*) if and only if (*f*(*F*_1_), *A*_1_) is a generalized soft ideal of (*G*, *B*). And (*F*_1∗_, *A*_1_×_∗_ *A*)≃_*G*_(*f*(*F*_1_)_∗_, *A*_1_×_∗_ *B*), where (*F*_1∗_, *A*_1_×_∗_ *A*) is a generalized soft quotient ring of (*F*, *A*) with respect to (*F*_1_, *A*_1_), and (*f*(*F*_1_)_∗_, *A*_1_×_∗_ *B*) is a generalized soft quotient ring of (*G*, *B*) with respect to (*f*(*F*_1_), *A*_1_).

**Proof**

(1) Let (*F*_1_, *A*_1_), (*F*_2_, *A*_2_) ∈ SS((*F*, *A*)). If (*f*(*F*_1_), *A*_1_) = (*f*(*F*_2_), *A*_2_), that is, for all *α* ∈ *A*_1_, ∃*β* ∈ *A*_2_ such that *f*(*F*_1_)(*α*) = *f*(*F*_2_)(*β*) and for all *b* ∈ *A*_2_, ∃*a* ∈ *A*_1_ such that *f*(*F*_2_)(*b*) = *f*(*F*_1_)(*a*), but by Lemma 37, we have *F*_1_(*α*) = *F*_2_(*β*) and *F*_2_(*b*) = *F*_1_(*a*), which means that (*F*_1_, *A*_1_) = (*F*_2_, *A*_2_). Thus it is injection. Let (*F*_1_, *A*_1_) ∈ SS((*F*, *A*)); we have that, for all *α* ∈ *A*_1_, *F*_1_(*α*) ≤ *F*(*α*); then its homomorphism image *f*(*F*_1_(*α*)) ≤ *f*(*F*(*α*)) = *G*(*g*(*α*)), and so $\left(f(F_{1}),A_{1}){\tilde{\leq }}_{G}(G,B\right)$; that is, (*f*(*F*_1_), *A*_1_) ∈ SS((*G*, *B*)). Let (*f*(*F*_1_), *A*_1_) ∈ SS((*G*, *B*)), we have that, for all *α* ∈ *A*_1_, ∃*g*(*α*) ∈ *B* such that *f*(*F*_1_(*α*)) ≤ *G*(*g*(*α*)). So we can define *F*_1_(*α*) = {*x* ∈ *R* | *f*(*x*) ∈ *f*(*F*_1_(*α*))}; then for all *α* ∈ *A*_1_, *F*_1_(*α*) ≤ *F*(*α*) and for all *β* ∈ *A*′, ∃*α* ∈ *A*_1_, Ker⁡*f*(*F*(*β*)) = *F*′(*β*)⊆*F*_1_(*α*). So $\left(F_{1},A_{1})\tilde{\leq }(F,A\right)$ and $\left(F^{\prime },A^{\prime }){\tilde{\subset }}_{C}(F_{1},A_{1}\right)$; that is, (*F*_1_, *A*_1_) ∈ SS((*F*, *A*)). Thus it is surjection. Therefore, we make (*F*_1_, *A*_1_) correspond to (*f*(*F*_1_), *A*_1_), which is a bijective mapping between SS((*F*, *A*)) and SS((*G*, *B*)).

(2) $\left(F_{1},A_{1})\tilde{⊲}(F,A)\Leftrightarrow F_{1}(\alpha )⊲F(\alpha \right)$  (for all *α* ∈ *A*_1_)⇔ (by Lemma 37) *f*(*F*_1_(*α*))⊲*f*(*F*(*α*)) = *G*(*g*(*α*)) (for all *α* ∈ *A*_1_, ∃*g*(*α*) ∈ *B*) $\Leftrightarrow (f(F_{1}),A_{1}){\tilde{⊲}}_{G}(G,B)$. By Lemma 37, we have that, for all (*α*, *α*) ∈ *A*_1_×_∗_ *A*, ∃(*α*, *g*(*α*)) ∈ *A*_1_×_∗_ *B* such that *F*_1∗_(*α*, *α*)≅*f*(*F*_1_)_∗_(*α*, *g*(*α*)), which means that (*F*_1∗_, *A*_1_×_∗_ *A*)≃_*G*_(*f*(*F*_1_)_∗_, *A*_1_×_∗_ *B*).

**Lemma 55 (see [24] second isomorphism theorem)**

Let *S* be a subring and let *H* be an ideal of *R*, respectively. Then *S* + *H* = {*x* + *y* | *x* ∈ *S*, *y* ∈ *H*} is a subring of *R*, *S*∩*H* is an ideal of *S*, and a mapping *f* : *S*/(*S*∩*H*)→(*S* + *H*)/*H*, for *x* + (*S*∩*H*) ∈ *S*/(*S*∩*H*), *f*(*x* + (*S*∩*H*)) = *x* + *H*, is an isomorphism. That is, *S*/(*S*∩*H*)≅(*S* + *H*)/*H*.

**Theorem 56 (third soft isomorphism theorem)**

Let (*F*, *A*) be a soft ring and (*G*, *B*) be an idealistic soft ring over *R*.

1. (*H*, *C*) = (*F*, *A*)+(*G*, *B*) is a soft ring over *R*, and (*G*, *B*) is a generalized soft ideal of (*H*, *C*);
2. (*K*, *D*) = (*F*, *A*)∩_*R*_(*G*, *B*) is a generalized soft ideal of (*F*, *A*);
3. For all (*b*, *c*) ∈ *B*×_∗_ *C*, there exists (*d*, *a*) ∈ *D*×_∗_ *A* such that a mapping *f* : *G*_∗_(*b*, *c*) → *K*_∗_(*d*, *a*), with respect to *x* + *K*(*d*) ∈ *K*_∗_(*d*, *a*) and *f*(*x* + *K*(*d*)) = *x* + *G*(*b*), is an isomorphism, where *x* ∈ *F*(*a*). That is, (*G*_∗_, *B*×_∗_ *C*)≃_*G*_(*K*_∗_, *D*×_∗_ *A*), where (*G*_∗_, *B*×_∗_ *C*) is a generalized soft quotient ring of (*H*, *C*) with respect to (*G*, *B*), and (*K*_∗_, *D*×_∗_ *A*) is a generalized soft quotient ring of (*F*, *A*) with respect to (*K*, *D*).

**Proof**

(1) By given conditions, we observe that, for all *α* ∈ *A*, *F*(*α*) ≤ *R* and for all *β* ∈ *B*, *G*(*β*)⊲*R*. By Lemma 55, we have that, for all (*α*, *β*) ∈ *C*, *F*(*α*) + *G*(*β*) = *H*(*α*, *β*) ≤ *R*; then $(H,C)\tilde{<}R$. And, it is obvious that, for all *β* ∈ *B*, ∃(*α*, *β*) ∈ *C* such that *G*(*β*)⊲*H*(*α*, *β*). Hence, $\left(G,B){\tilde{⊲}}_{G}(H,C\right)$.

(2) By Definition 6, we know that, for all *α* ∈ *D* = *A*∩*B* ≠ *∅*, *K*(*α*) = *F*(*α*)∩*G*(*α*). Since *F*(*α*) ≤ *R*, *G*(*α*)⊲*R*, then by Lemma 55, we have for all *α* ∈ *D*, *F*(*α*)∩*G*(*α*) = *K*(*α*)⊲*F*(*α*). Therefore (*K*, *D*) is a generalized soft ideal of (*F*, *A*).

(3) By Definition 48, we have that, for all *β* ∈ *B*, ∃(*α*, *β*) ∈ *C* such that *G*_∗_(*α*, *β*) = {*x* + *G*(*β*) | *x* ∈ *H*(*α*, *β*)}, and we observe that, for all *α* ∈ *D* = *A*∩*B*, ∃*α* ∈ *A* such that *K*_∗_(*α*, *α*) = {*y* + *F*(*α*) | *y* ∈ *K*(*α*)}, where *G*(*β*)⊲*H*(*α*, *β*) and *K*(*α*)⊲*F*(*α*). By Lemma 55, we have that, for all (*α*, *β*) ∈ *B*×_∗_ *C*, ∃(*α*, *α*) ∈ *D*×_∗_ *A*, such that a mapping *f*, with respect to *x* + *K*(*α*) ∈ *K*_∗_(*α*, *α*) and *f*(*x* + *K*(*α*)) = *x* + *G*(*α*), is an isomorphism, where *x* ∈ *F*(*α*). That is, *G*_∗_(*α*, *β*)≅*K*_∗_(*α*, *α*), which means that (*G*_∗_, *B*×_∗_ *C*)≃_*G*_(*K*_∗_, *D*×_∗_ *A*) by Definition 50.

## 6. Conclusions

In this paper, concepts of soft congruence relations are presented. Two types of soft congruence relations that are soft congruence relation over a ring and soft congruence relation of a soft ring are defined in a natural way. Then we study relations among soft congruence relations and homomorphisms, soft ring homomorphisms. Also, we obtain a one-to-one correspondence between soft congruence relations and idealistic soft rings and a one-to-one correspondence between soft congruence relations and soft ideals. In particular, we established the first, second, and third soft isomorphism theorems, respectively. In the light of these results, our future work on this topic will be focused on applying soft congruence relations to fuzzy information over rings.
